# Enhancing Rubber Industry Wastewater Treatment through an Integrated AnMBR and A/O MBR System: Performance, Membrane Fouling Analysis, and Microbial Community Evolution

**DOI:** 10.3390/membranes14060130

**Published:** 2024-06-05

**Authors:** Ishanka Prabhath Wimalaweera, Yuansong Wei, Fumin Zuo, Qihe Tang, Tharindu Ritigala, Yawei Wang, Hui Zhong, Rohan Weerasooriya, Shameen Jinadasa, Sujithra Weragoda

**Affiliations:** 1 State Key Joint Laboratory of Environmental Simulation and Pollution Control, Research Center for Eco-Environmental Sciences, Chinese Academy of Sciences, Beijing 100085, China; ishankapra@mails.ucas.ac.cn (I.P.W.); fmzuo_st@rcees.ac.cn (F.Z.); 2208401014@st.gxu.edu.cn (Q.T.); tharindu@bewg.net.cn (T.R.); wangyawei@rcees.ac.cn (Y.W.); zhhui@rcees.ac.cn (H.Z.); 2 Laboratory of Water Pollution Control Technology, Research Center for Eco-Environmental Sciences, Chinese Academy of Sciences, Beijing 100085, China; 3 University of Chinese Academy of Sciences, Beijing 100049, China; 4 China-Sri Lanka Joint Research and Demonstration Center for Water Technology, Ministry of Water Supply, Meewathura, Kandy 20400, Sri Lanka; skwera7@gmail.com; 5 National Institute of Fundamental Studies, Hanthana Road, Kandy 20000, Sri Lanka; rohan.we@nifs.ac.lk; 6 Department of Civil Engineering, University of Peradeniya, Kandy 20400, Sri Lanka; shamj@eng.pdn.ac.lk; 7 School of Engineering and Technology, Central Queensland University, Bundaberg, QLD 4670, Australia; 8 National Water Supply and Drainage Board, Kandy 20800, Sri Lanka

**Keywords:** natural rubber industrial wastewater, membrane bioreactor, membrane fouling, ceramic membrane, integrated AAO process, simultaneous N and S removal

## Abstract

This study explores the effectiveness of an integrated anaerobic membrane bioreactor (AnMBR) coupled with an anoxic/oxic membrane bioreactor (A/O MBR) for the treatment of natural rubber industry wastewater with high sulfate, ammonia, and complex organic contents. This study was conducted at the lab-scale over a duration of 225 days to thoroughly investigate the efficiency and sustainability of the proposed treatment method. With a hydraulic retention time of 6 days for the total system, COD reductions were over 98%, which reduced the influent from 22,158 ± 2859 mg/L to 118 ± 74 mg/L of the effluent. The system demonstrates average NH_3_-N, TN, and total phosphorus (TP) removal efficiencies of 72.9 ± 5.7, 72.8 ± 5.6, and 71.3 ± 9.9, respectively. Despite an average whole biological system removal of 50.6%, the anaerobic reactor eliminated 44.9% of the raw WW sulfate. Analyses of membrane fouling revealed that organic fouling was more pronounced in the anaerobic membrane, whereas aerobic membrane fouling displayed varied profiles due to differential microbial and oxidative activities. Key bacterial genera, such as Desulfobacterota in the anaerobic stage and nitrifiers in the aerobic stage, are identified as instrumental in the biological processes. The microbial profile reveals a shift from methanogenesis to sulfide-driven autotrophic denitrification and sulfammox, with evidence of an active denitrification pathway in anaerobic/anoxic conditions. The system showcases its potential for industrial application, underpinning environmental sustainability through improved wastewater management.

## 1. Introduction

The global rubber industry plays a pivotal role, supplying critical materials to various sectors such as automotive, manufacturing, and healthcare, thus significantly contributing to the economies and technological growth of numerous countries such as those in the Asia–Pacific region, including China and India [1]. However, the process of rubber manufacturing generates a substantial volume of wastewater laden with a diverse array of pollutants including organic compounds, ammonia, sulfate, and various chemical additives. Rubber wastewater is an iconic representation of high-nitrogen (N) and -sulfur (S) wastewater, illustrative of the complicated difficulties posed by many industrial effluents [2]. The release of inadequately treated wastewater into natural environments poses serious risks on and impacts aquatic ecosystems and biodiversity because it contributes to eutrophication, toxic algal blooms, and oxygen deprivation [3]. As environmental concerns mount, the importance of adopting sustainable practices in wastewater management is underscored, not only to mitigate ecological harm but also to comply with increasingly stringent global regulations (see Supplementary Material for standards for industrial wastewater discharge into centralized treatment plants or public sewers in China and Sri Lanka [4,5]. The treatment of natural rubber industry wastewater (NRIWW) presents significant challenges due to its intricate composition, the propensity for biological process inhibition, high fouling potential, and impact on microbial community dynamics [6]. These issues underscore the need for innovative and resilient treatment methodologies that can adapt to the unique and varying characteristics of rubber industry wastewater.

There have been extensive studies on both the efficacy and shortcomings of conventional treatment methods for NRIWW. While bioaugmentation and bioremediation are often suggested as effective, there is a scarcity of focused research on the performance of integrated biological treatment processes [7]. The rubber industry frequently employs several conventional wastewater treatment systems, such as facultative ponds, anaerobic filter beds, rotating biological contactors, aerated lagoons, upflow anaerobic sludge blankets (UASBs), and oxidation ditches. These traditional methods come with a host of limitations, including suboptimal efficiency, longer hydraulic retention time (HRT), extensive spatial needs, and considerable energy demands and often exceed discharge limits [7]. Integrated processes enhance the overall effectiveness of wastewater treatment by leveraging the strengths and mitigating the weaknesses of each individual method, leading to a more efficient system. Integrating anaerobic, anoxic, and aerobic processes in wastewater treatment offers enhanced efficiency, characterized by shorter HRTs, high resilience to load fluctuations, and reduced operational costs [8,9,10]. The development of an integrated anaerobic–aerobic system specifically for rubber industry wastewater was initiated by researchers using a two-stage UASB paired with a downflow hanging sponge (DHS) system. The DHS system significantly reduces power usage by 97% and the production of excess sludge by 98%. The overall system achieves a COD removal efficiency of 95.7% at an organic loading rate of 0.8 kg COD/m^3^⋅day, with the two-stage UASB removing over 80% of sulfates and the DHS effectively oxidizing residual organic material and sulfides [4,11,12]. Research on integrated aerobic–anaerobic reactors has primarily been focused on the removal of organic and nitrogenous waste, with many systems successfully eliminating over 80% of contaminants [6]. However, there is room for improvement in these systems’ capacity to handle organic loads, as evidenced by systems that, despite handling higher loads, fail to meet regulatory standards.

By integrating semipermeable membrane processes with biological wastewater treatment, MBR systems, particularly with ultrafiltration (UF), this membrane is recognized for its ability to separate contaminants efficiently, utilizing minimal chemicals and having the ability for continuous commercial operations due to its moderate pore size, which allows for low-pressure operation and less energy use, thereby allowing for a more concentrated biodegradable process and allowing for higher solid retention times (SRTs) and enhanced organic decomposition. This increased efficiency not only enables the treatment of higher volumes of organic pollutants but also ensures that the treated wastewater meets and often exceeds the required discharge standards [13]. Studies have also proven that MBR systems with UF flat-sheet membranes alone could treat nitrogen in high-strength skim latex wastewater greater than 60% while archiving COD removal more than 96% [14]. Despite its advantages, membrane fouling remains a significant challenge, resulting in increased operational costs, higher energy requirements, shorter membrane lifespan, and a greater need for frequent cleaning. During MBR applications in the rubber industry, a notable decline in flux occurs due to the accumulation of dissolved organic matter on the membrane surface, leading to membrane fouling or concentration polarization [15,16]. The presence of inorganic foulants, including sulfate and phosphate ions, is a prevalent issue in NRIWW processing, which contributes to membrane fouling through chemical precipitation of the inorganic species as well as the biological formation of inorganic–organic complexes [17].

With the intention of reducing the footprint and membrane fouling, lowering operating costs and achieving higher effluent quality in the treatment of industrial effluent with elevated levels of NH_4_^+^-N, PO_4_^3−^, COD, and SO_4_^2−^, simultaneous nutrient removal in an integrated system has been the current focus of research [17]. Huang et al. studied the anaerobic–anoxic–oxic (AAO)–MBR–biological aerated filter (BAF)–O_3_ process for treating high-organic wastewater with high ammonia nitrogen, and under optimal conditions, the removal efficiencies of COD, NH_4_^+^–N, TN, and TOC were 94.50%, 99.13%, and 78.21% [18]. The AAO-MBR system, employing controlled aeration, improved nutrient elimination and mitigated membrane fouling through the enlargement of sludge aggregates. Achieving optimal performance at a membrane flux of 30 L per square meter per hour, this system successfully removed up to 81.5 ± 6.1% of TN and 96.7 ± 2.1% of TP [19]. During another study, the AAO + MBR technique held potential for enhanced nitrogen and phosphorus elimination in conventional wastewater treatment facilities. The efficiency of removing TN and TP was consistently sustained at 80–90%, while the effluent’s COD levels were kept below 30 mg/L [20]. Zuo et al. conducted a study on the AAO-MBR system to efficiently extract nutrients from wastewater in conditions of low dissolved oxygen. Their research reported removal efficiencies of 91% for COD, 98% for NH_3_-N, 88% for PO_4_^3−^-P, and 93% for total inorganic nitrogen [21]. Research on AAO–MBR systems lacks analysis of the simultaneous removal of major pollutants including NH_3_, TN, COD, SO_4_^2−^, and PO_4_^3−^; membrane fouling analysis; and a full investigation and analysis of the microbial community and metabolic pathways. Operating costs of implementing the proposed MBR technology at full scale can be estimated by considering factors such as initial capital investment, energy recovery potential, sludge handling strategies, and operational efficiencies. While the life cycle costs for AnMBR + aerobic MBR (ArMBR) systems are higher by 17% and 23% compared to conventional activated sludge systems (CAS) and AnMBR + CAS, respectively, ArMBR-based post-treatments are attractive alternatives, especially for applications requiring high-quality effluent and water reuse [22,23]. Supplementary Material includes (Table S2) the comprehensive cost breakdown, categorized into capital and operating and maintenance (O&M) expenses, for various treatment configurations such as CAS, ArMBR, and AnMBR (including combined setups like AnMBR with ArMBR and AnMBR with CAS), specifically focusing on nutrient removal. Integrating AnMBR with ArMBR offers benefits such as improved energy recovery potential and enhanced nutrient removal efficiency. This integration leads to better sludge management and optimized energy recovery, reducing the environmental impact and promoting a more circular economy.

This study aims to investigate the performance of an integrated AnMBR and A/O MBR system with a focus on the simultaneous removal of NH_3_, TN, COD, SO_4_^2−^, and PO_4_^3−^ from NRIWW, combined with membrane fouling behavior and microbial community analysis. This study conducted a long-term investigation of the system performance and the membrane fouling behavior under the influence of actual wastewater from the rubber industry, which is considered to be high-strength wastewater with elevated NH_3_ and SO_4_^2−^ levels. Finally, the microbial community and metabolic pathways involved in nitrogen, sulfur, and organic matter removal were investigated at each reactor in this system.

Ceramic membranes, used in this study due to their exceptional performance, offer advantages such as resistance to acids and alkalis, high thermal stability, excellent chemical stability, robust mechanical strength, and ease of cleaning and regeneration [24]. These attributes have led to their increased use in various fields, including medical, biological, and environmental applications. Over the past decade, their application in industrial and urban wastewater as well as drinking water treatment has seen rapid growth. Ceramic membranes outperform hollow-fiber membranes in terms of pore size, water production, and overall durability and resistance to aggressive chemicals [25]. Moreover, ceramic membranes can handle high SS concentrations, which is particularly beneficial for rubber industrial wastewater that often has SS concentrations. These facts make ceramic membranes ideal for treating industrial wastewater with high levels of ammonia and sulfate.

## 2. Materials and Methods

### 2.1. The Natural Rubber Industry’s Raw Wastewater and Pretreatment Procedure

The wastewater from concentrated latex manufacturing is regarded as particularly hazardous, mainly due to the use of high ammonia levels to stabilize the natural latex and the substantial use of acidic chemicals in coagulation processes [22,23]. At a centrifuged latex manufacturing facility in Mawanella, Sri Lanka (7°15′22.2″ N; 80°26′27.9″ E), where natural latex is produced, wastewater samples were gathered. Concentrated latex was created by centrifuging natural rubber at 7000 to 10,000 rpm. The rubber was then ammoniated to preserve it. Sulfuric acid was then used to coagulate the less concentrated latex part, known as skim latex, to create skim rubber. Once this skim rubber dried, it was made into sheets. The effluent water known as skim serum water was discarded as effluent after the coagulum was extracted. The factory produces raw wastewater that is separated into wash water from milled rubber sheets, acidified effluent, skim serum, and wastewater that is used to clean the centrifuge machine. The pretreatment stage plays a crucial role in the effective treatment of NRIWW by removing materials that are inhibitory to downstream processes. NRIWW contains small amounts of uncoagulated latex and serum liquid, along with high concentrations of proteins, lipids, carotenoids, carbohydrates, sugars, and various organic and inorganic salts. Additionally, the wastewater is characterized by significant total, suspended, and dissolved solids, which can have severe ecological impacts. Pretreatment is usually focused on the removal of suspended solids through numerous physical and chemical processes. By targeting and removing high levels of SS, organic matter, and heavy metals early in the treatment process, pretreatment ensures that subsequent secondary and tertiary treatments operate more efficiently and effectively.

Raw rubber wastewater was pretreated using magnetic seed coagulation (MSC) before being introduced into a biological system. Details of the pretreatment system can be found in our earlier publication [26]. The MSC process, utilizing polyaluminum chloride (PAC), anionic polymer (polyacrylamide—PAM), and magnetic seeds (ferric oxide (Fe_3_O_4_)), has proven to be a cost-effective pretreatment method for NRIWW. When enhanced with calcium hydroxide (Ca(OH)_2_), this method significantly improves the removal efficiency of turbidity, COD, and TSS by 95%, 56%, and 64%, respectively. The organic components in NRIWW interact with the magnetic seeds, forming Fe–OH/Fe–OH⁺ linkages through surface complexing and hydrogen bonding. Additionally, magnetic seeds act as adsorbents, enhancing the development of denser and larger magnetic flocs.

The feeding-water characteristics of the AnMBR after MSC pretreatment are listed in Table 1.

### 2.2. Experimental Setup

**Figure 1:**
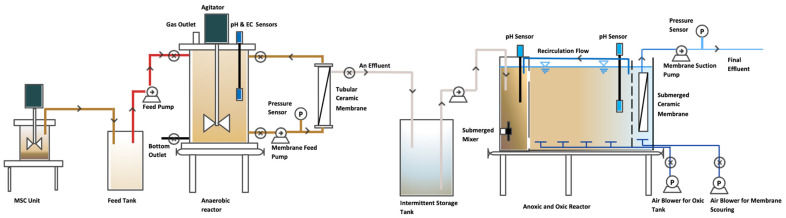
Schematic diagram of the AnMBR and anoxic/oxic MBR system.

The AnMBR was a cylindrical tank with an effective volume of 15 L (diameter = 230 mm, height = 450 mm), and a single tank was partitioned into three sections that contained anoxic, oxic, and submerged membranes (total tank dimensions: length: 390 mm, width: 100 mm, liquid height: 400 mm) as anoxic and oxic reactors. Setup was operated at room temperature. The external tubular membrane was made of ceramic (yttria-stabilized zirconia) with a nominal pore size of 0.1 μm, and a total area of 0.11 m^2^ (Hefei Shijie Membrane Engineering Co., Ltd., Hefei, China) was coupled with the AnMBR. Similar to the anaerobic membrane, the aerobic membrane also contained ceramic, yttria, and zirconia with a nominal pore size of 0.1 μm. However, the aerobic membrane was a submerged membrane with a flat-sheet configuration. External ceramic membranes in AnMBRs offer significant advantages, including better maintenance accessibility, effective backwashing, and greater control over hydraulic conditions like transmembrane pressure and cross-flow velocity. These features enhance operational efficiency and reduce fouling caused by extracellular polymeric substances (EPSs) and inorganic substances produced from biological reactions (i.e., sulfur from sulfammox and SDAD processes), making them ideal for high-load wastewater treatment. Moreover, they allow for modular upgrades and handle higher solids concentrations, which is crucial for anaerobic digestion processes [27]. Conversely, submerged membranes in anoxic/oxic MBRs are directly immersed in the mixed liquor, promoting seamless integration with biological processes, energy efficiency as the aeration serves both to provide oxygen and to scour the membranes, lower circulation needs, and reduced operational complexity with fewer mechanical components [28]. A coarse air bubble diffusing arrangement was also fixed at the bottom of the submerged membrane to reduce membrane fouling (Figure 1). Peristaltic pumps (LanN1, YZ1515x, SHENCHEN, Baoding, China) were used to feed the pretreated wastewater into the AnMBR and then AnMBR effluent to the anoxic tank, as shown in Figure 1. The ceramic membrane were equipped with pressure gauges to monitor transmembrane pressure. Another two types of peristatic pumps (ALLEDOSIEREN, C series, Sichuan, China, and LanM3, YZ1515x Pump, SHENCHEN, Baoding, China) were used to feed the membrane in the AnMBR and to draw the final effluent through the membrane in the oxic tank. The AnMBR contained sensors and a gas outlet, which were used to track the temperature, pH, and EC of the water treatment system.

### 2.3. Operation of the Bioreactor

Samples of NRIWW and seed sludge were taken from the same latex production plant in Mawanella, Sri Lanka. The factory had an aerobic and anaerobic suspended growth wastewater treatment system in place. The AnMBR was run for 225 days in this experiment, split into four phases according to loading rates and influent quality. Monitoring and analysis of the concentrations of the main pollutants in the influent, anaerobic effluent, and final oxic MBR effluent were performed in order to establish the process and optimize its parameters for the relevant biological processes in the reactor system. During these times, the reactor’s influent loading strategy was sequential. The system was fully fed with actual wastewater from day 71 onwards. Details of the system’s loading conditions are shown in Table 2.

The MBR system utilized a time-based backwashing approach designed to optimize pump longevity, cost efficiency, and energy use. Daily backwashing was performed using 1 L of permeate for 60 s. The anaerobic membrane received backwashing at 150 kPa and 150 L/m^2^/h, whereas the aerobic membrane was backwashed at a flux of 15 L/m^2^/h. Upon TMP reaching 85 kPa for the anaerobic membrane and 40 kPa for the aerobic membrane, ex situ chemical cleaning was conducted. This process included a sequence of rinsing with water, cleaning with a 500 ppm NaOCl solution, and cleaning with a citric acid (500 ppm) solution [29], each followed by a water soak for 4 h. The pH values of the NaOCl and citric acid solutions were within the ranges of 10.0–10.5 and 3.5–4.0, respectively. Both NaOCl and citric acid soaking was conducted for 2 h each. All cleaning solutions were collected for analysis of the cleaning efficacy and fouling characteristics.

### 2.4. Analytical Methods

#### 2.4.1. Analysis of Chemical and Physical Parameters

Each and every parameter was analyzed using the usual procedure (APHA, 2017). Utilizing a HACH HQ 40d multiparameter water quality analyzer (Hach, Loveland, CO, USA), pH and conductivity were determined. After the supernatant samples were run through a 0.45 μm membrane filter (GD/XP Syringe Filters, Whatman, Neots, UK), the dissolved components were examined. Using an ultraviolet spectrophotometer (HACH DR 6000), the COD, S^2−^, and alkalinity were measured. Dissolved phosphorous was measured using the ascorbic acid method. The NH_4_^+^-N was quantified using the phenolate technique. An ion chromatography system (IC, ICS 1000, Dionex, Sunnyvale, CA, USA) was used to quantify the concentrations of SO_4_^2−^, NO_3_^−^, and NO_2_^−^. With the use of a prefabricated tube reagent (HACH, USA), COD and alkalinity were assessed. Using a TOC analyzer (LCPH/CPN, Shimatzu, Kyoto, Japan), TOC and TN were determined. Organic foulants on the membrane were analyzed by a three-dimensional fluorescent excitation–emission matrices analyzer (3D-EEM, F-7000, Hitachi, Tokyo, Japan). The morphology of the foulants and membranes was observed by field emission scanning electron microscopy (FE-SEM, HITACHI SU8020, Hitachi, Japan) and a Hitachi S-3000N scanning electron microscope (SEM, Hitachi, Japan) equipped with energy dispersive spectroscopy (EDS), and EDS mapping was applied to investigate the elemental distribution assessment across the membrane surfaces. IBM STATISTICS SPSS was used to evaluate all of the data.

#### 2.4.2. Microbiological Examination

On Days 34, 62, 90, 119, 140, 181, and 220, respectively, samples were taken from the mixed liquid in order to evaluate the bacterial and archaeal communities. DNA was extracted from the mixed liquor using the MP Biomedicals, Solon, OH, USA, FAST DNA Spin Kit for Soil. After that, DNA samples were gathered and sent for examination to Majorbio Bio-Pharm Technology Co., Ltd. in Shanghai, China. To evaluate the bacterial and archaeal populations, PCR amplification of the 16S rRNA genes was carried out using the primer pairs 515F/806R and 515F/806L. At Shanghai, China’s, Shanghai Majorbio Bio-pharm Technology Co., Ltd. sequencing facility, paired-end Illumina sequencing (Illumina MiSeq, San Diego, CA, USA) was carried out. An online platform for processing raw data was developed by Sangon Co., Ltd., Shanghai, China (Project No. MJ-M-20230610061). The Kyoto Encyclopedia of Genes and Genomes (KEGG) database was used for functional annotation and classification. Genes associated with carbon, sulfur, and nitrogen metabolism pathways and enzyme activities were identified by looking at the KEGG orthology (KO) numbers. This allowed for a more comprehensive analysis of the functions and transformation processes of the genes. The structural integrity and alpha and beta diversity of microbial communities were evaluated using the cloud analysis tool available online at https://cloud.majorbio.com/, accessed on 28 March 2024. The online analytical platform http://cloud.biomicroclass.com/CloudPlatform, accessed on 29 March 2024 was employed to assess the relationship between microbial populations and environmental factors.

### 2.5. Calculations

The fluorescence index (FI) in the 3D EEM analysis and nitrite accumulation ratio (NAR) [30] were calculated using Equations (1) and (2), respectively.

(1) $$\mathrm{FI}=\frac{{\;I}_{450}\;}{I_{500}}$$

where I_450_ and I_500_ are the emission intensities at 450 nm and 500 nm, respectively, when excited at 370 nm.

(2) $$\mathrm{NAR}=\frac{\left(\Delta {{\mathrm{NO}}_{2}}^{-}-N\right)}{\left(\Delta {{\mathrm{NO}}_{2}}^{-}-N+\Delta {{\mathrm{NO}}_{3}}^{-}-N\right)}$$

Typical theoretical equations commonly applied in previous research for membrane fouling behavior analysis were followed. For transmembrane pressure, flux, and permeability, Equations (3)–(5) were used [29,31].

(3) $$\mathrm{TMP}=\frac{\;\left(T1+T2\right)\;}{2}$$

where T1—inlet pressure (kPa) of the membrane, and T2—outlet pressure (kPa) of the membrane.

(4) $$J=\frac{\;V}{A\Delta t}$$

(5) J=K⋅ΔP

where J—permeate flux (Lm^−2^h^−1^), V—permeate volume (L), A—effective membrane filtration area (m^2^), t—unit filtration time (h), K—permeability (L/m^2^·h·bar), and ΔP—transmembrane pressure difference (bar).

## 3. Results

### 3.1. Perforemance of Pollutants Removal

The AnMBR and A/O MBR system’s operational evaluation over 225 days, segmented into four distinct loading phases, provided in-depth insights into the treatment of rubber industry wastewater.

#### 3.1.1. COD/TOC Removal

**Figure 2:**
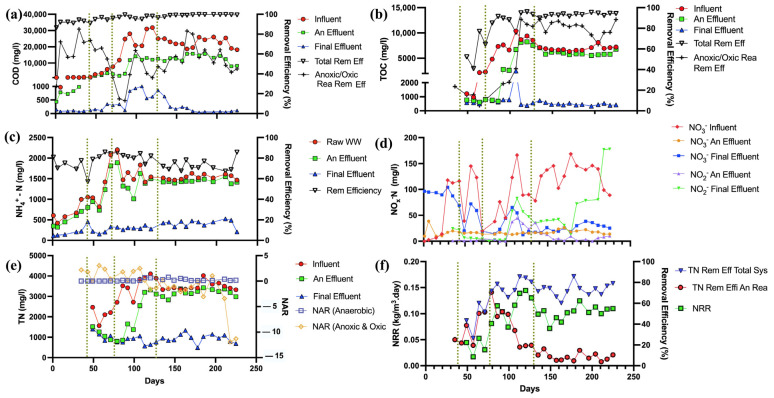
Performance of AnMBR—A/O MBR system in organic and nitrogen compound removal: (**a**) COD level variation with removal efficiencies, (**b**) TOC level variation with removal efficiencies, (**c**) NH_4_^+^-N level variation with removal efficiencies, (**d**) NOx level variation, (**e**) TN variation and NAR variation, (**f**) NRR variation and TN removal efficiencies of the system.

The initial phase, with its moderate influent COD and TOC levels, saw the system efficiently beginning the treatment process with a COD removal efficiency of 91.5 ± 2.5 (Figure 2a). As the loading conditions intensified in the subsequent phases, the system not only coped with the heightened organic load but also improved its performance, achieving an average COD removal efficiency of up to 99.4 ± 0.4% in the final phase. This high level of COD removal was consistent with the stringent discharge standards set by both China and Sri Lanka, indicating that the treated effluent was well within the regulatory limits for organic pollutants. Such compliance is particularly noteworthy given the complexity of the influent characteristic of rubber industry wastewater. Throughout all phases, the TOC concentrations in the final effluent maintained consistently low levels, which reflects the AnMBR and A/O MBR system’s capability in achieving stable TOC removal. Effluent TOC levels displayed the removal efficiencies of 86.8 ± 20.8, 58.7 ± 15.5, 89.4 ± 6.1, and 93.4 ± 6.1% at each phase (from 1 to 4), respectively. The fluctuations in influent TOC, particularly the spikes in the latter phases, did not deter the system from delivering high-quality effluent (Figure 2b).

The observed reduction in COD and TOC across all phases indicates robust organic matter degradation, which validates the results from similar studies such as that by Z. Wang et al. [32], which demonstrated the role of enzymatic processes in breaking down complex organic molecules in anaerobic reactors, leading to a decrease in COD as the organic matter was transformed into simpler forms. In this study, also, the significant reductions in COD during the anaerobic phase signaled efficient conversion of organic matter into VFAs and biogas. H. Liu et al. [33] highlighted the critical role of organic carbon as an energy source for denitrification in anoxic conditions. In alignment with this, the consumption of organic carbon during our study’s anoxic phase led to further decreases in COD, affirming the hypothesis that residual organic matter from anaerobic digestion supports denitrification processes. The Anoxic and oxic reactor displayed COD removal efficiencies of 63.5 ± 18.7, 55.3 ± 13.4, 38.9 ± 16.5, and 58.3 ± 12.8% at each phase (from 1 to 4), respectively. In the aerobic phase, the results mirrored the expected nitrification reaction dynamics while further reducing COD.

#### 3.1.2. NH_3_/TN Removal

NH_3_ removal of the total system averaged from 70.8% in Phase 1 to 72.9% by Phase 4. The main NH_3_ removal mechanisms were the sulfammox process, along with sulfide-driven autotrophic denitrification (SDAD) for anaerobic and anoxic conditions and nitrification for the oxic environment (Figure 2c). The NAR in the anoxic/oxic reactor fluctuated, indicating varying degrees of nitrite accumulation, with averages ranging from 2.00 in Phase 1 to −3.24 in Phase 4, reflecting the dynamic nature of the biological processes [34,35]. Furthermore, the nitrogen removal rate (NRR) in the system, indicative of the kinetics of nitrogen removal processes, showed a marked efficacy. The system’s performance, with an NRR starting at 0.071 kg TN/m^3^/day in Phase 1 and increasing to 0.106 kg TN/m^3^/day by Phase 4 (Figure 2f), underscores the capability of the AnMBR and A/O MBR to handle the nitrogen load effectively. In the anaerobic reactor, the NAR was consistently low or near zero (Figure 2e), which, along with the observed decrease in NO_X_ concentrations, supports efficient denitrification, with minimal nitrite accumulation. This process was efficient enough to result in the anaerobic reactor showing an improvement in NO_X_ removal efficiency from an average of 39.53% in Phase 1 to 85.49% by Phase 4, indicating a substantial conversion of nitrate to nitrogen gas [35]. The oxic reactor’s role was crucial in ammonia conversion to nitrate, evidenced by the increase in NO_X_ concentrations (Figure 2d). This suggests the significant role of ammonia-oxidizing bacteria (AOB) and nitrite-oxidizing bacteria (NOB) in the nitrification process [36]. However, the higher NAR in the oxic reactor suggests incomplete nitrification, with a potential for nitrite accumulation that might necessitate further optimization of the system [37].

Combining these observations with the TN removal efficiencies, which show a progressive decrease across the operational phases for the anaerobic reactor from 27.27% in Phase 1 to 8.55% in Phase 4, we can summarize by noting that the system faced challenges, likely due to the overburden of ammonia-oxidizing bacteria. Despite these challenges, the overall TN removal efficiency remained relatively high throughout the treatment process with an average of 72.82 ± 6.60 removal efficiency at full-strength wastewater loading.

#### 3.1.3. SO_4_^2−^-S Removal

**Figure 3:**
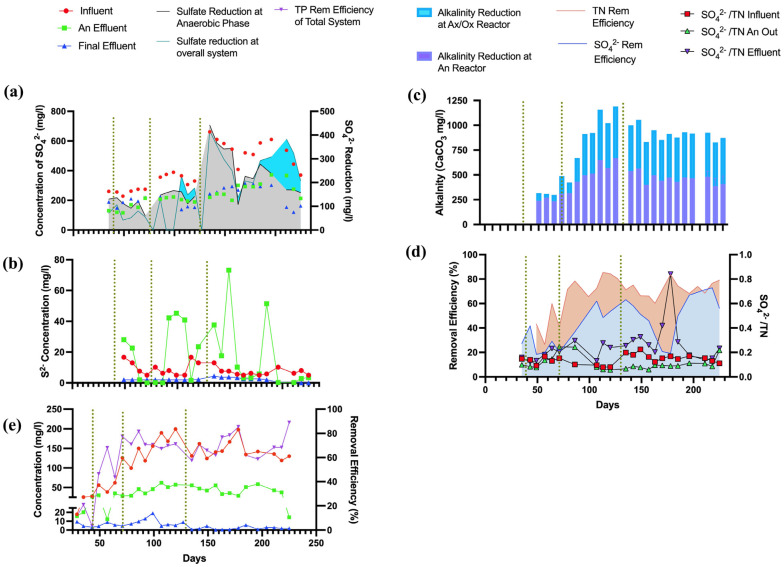
Performance of AnMBR—A/O MBR system in sulfur and TP compound removal: (**a**) SO_4_^2−^ level variation with removal efficiencies, (**b**) S^2−^ level variation, (**c**) alkalinity level reduction at anaerobic reactor and anoxic/oxic reactor, (**d**) TN, SO_4_^2−^ removal efficiencies and SO_4_^2−^/TN ratio variations, (**e**) TP level variation with removal efficiencies of the system.

In the anaerobic reactor, the average sulfate removal efficiency was observed to be 34.55% during Phase 1 (Figure 3a), indicating a robust initiation of the sulfate reduction process through the sulfammox process and presence of sulfate reduction bacteria (SRB). This process is further evidenced by the average reduction in alkalinity measured as 174.1 mg/L of CaCO_3_ in Phase 2 (Figure 3c), suggestive of acidogenic activity leading to a conducive environment for SRB. However, a decline in efficiency to 21.84% in Phase 2 implies a potential challenge in maintaining sulfate reduction, possibly due to operational conditions or influent variability. The SDAD mechanism, facilitated by denitrifying bacteria, was another process observed which uses both nitrate and sulfate as electron acceptors [38], thereby reducing the alkalinity in the system, with average reductions in the anoxic/oxic reactor measuring 38.675 mg/L CaCO_3_ in Phase 2 and increasing to 281.2 mg/L CaCO_3_ by Phase 3. The alkalinity consumption in these phases is reflective of the active denitrification and sulfate reduction, which is a clear demonstration of the versatility of microbial communities in the anoxic reactor. The autotrophic denitrification was evidenced by sulfide removal and the increase in alkalinity [39].

Biological removal of sulfide can be carried out by both aerobic oxidation and SDAD, by microorganisms using DO, or by nitrate from the aerobic nitrification zones for sulfide oxidation [39]. By Phase 3, the sulfide concentration peaks at an average of 27.26 mg/L, aligning with the higher sulfate removal efficiency reported in this phase in the anaerobic reactor (Figure 3b). As the process transitions into Phases 3 and 4, there is an evident increase in sulfate removal efficiencies to 48.29% and 49.62%, respectively, alongside a notable rise in alkalinity reduction in the anoxic/oxic reactor to averages of 281.2 mg/L and 193.27 mg/L of CaCO_3_. This suggests that conditions in later phases may have been optimized to support simultaneous denitrification and sulfate reduction in the presence of NO_3_^−^ in the anoxic reactor [40].

The sulfate/TN ratio further provides insight into the relative concentrations and removal efficiencies of these compounds. A decreasing ratio from the influent to the anaerobic reactor outlet suggests that nitrogen compounds are being removed more effectively than sulfate in the initial phases. However, an increasing ratio in the subsequent anoxic and oxic reactors, as seen with averages rising to 0.199 in Phase 2 and 0.304 in Phase 4, could indicate a relative abundance of nitrogen compounds in the effluent or a lower comparative removal rate of nitrogen compounds (Figure 3d). Reoxidation of elemental sulfur or sulfide into SO_4_^2−^ can readily take place via the SDAD process, resulting in a partial restoration of SO_4_^2−^ as well [41]. The steady average sulfate removal efficiency in the anaerobic reactor, at around 44.9% for the raw wastewater, reflects the importance of optimizing the conditions for sulfammox and SRB and enhancing the SDAD process for more effective sulfur compound transformations.

#### 3.1.4. Phosphorus Removal

The fluctuation in TP levels in anaerobic and anoxic stages may be linked to the release and assimilation of phosphorus, a balance between phosphorus release from dying cells, or endogenous respiration and uptake by polyphosphate-accumulating organisms (PAOs). The PAOs play a pivotal role in enhanced biological phosphorus removal processes, which are commonly utilized in modern wastewater treatment facilities. In the anaerobic reactor, phosphorus removal could be initiated through chemical precipitation, where phosphate ions in wastewater react with divalent cations such as calcium, magnesium, or iron, leading to the formation of insoluble phosphate salts that precipitate out of the solution [42,43]. This pathway is possible due to the use of lime in the pretreatment step. This dual nature of phosphorus dynamics suggests a complex interplay of processes that contribute to an average TP removal efficiency of 70.8% across the system (Figure 3e). In the oxic reactor, the presence of dissolved oxygen is crucial for the growth of aerobic bacteria. These microorganisms utilize organic carbon and incorporate phosphorus into their biomass, effectively reducing the TP concentration in the effluent. During the aerobic stage, PAOs utilize polyhydroxyalkanoates to support their growth and to replenish their stores of polyphosphate and glycogen. This process results in the overall removal of more phosphate from the wastewater during the aerobic phase than the amount released in the anaerobic phase, thereby ensuring a net reduction of phosphate in the treated effluent [43].

### 3.2. Mass Balance Analysis of Pollutants

In Phase 1 (0–40 days), the AnMBR initiated robust pollutant removal with a COD removal efficiency of 91.5% and NH_4_⁺-N removal efficiency of 70.8%. This phase laid the foundation for effective removal, with SO_4_^2−^-S removal efficiency at 34.6%. As the phases progressed, the system adapted and maintained high efficiencies, with Phase 2 (Days 41–70) seeing a slight dip in COD and NH_4_⁺-N removal efficiencies to 95.5% and 83.1%, respectively, likely reflecting the increased influent load. SO_4_^2−^-S removal efficiency also saw a decrease to 21.8%. By Phase 3 (Days 71–130), the treatment system demonstrated a rebound, with COD removal efficiency at 97.2% and NH_4_⁺-N removal efficiency increasing to 75.5%. This phase showed an improved SO_4_^2−^-S removal efficiency of 48.3%, indicating a more robust biological process taking hold. Phase 4 (Days 131–225) presented the system at its most efficient for COD removal, with an efficiency of 99.4%. NH_4_⁺-N and SO_4_^2−^-S removal efficiencies were also high at 72.9% and 49.6%, respectively. Throughout each phase, TP removal efficiency was noteworthy, averaging 70.8% across the system (Table 2). This suggests that chemical precipitation, alongside biological uptake, played a significant role in phosphorus management. The mass balance data underscore the system’s capacity to effectively adapt to varying influent loads and achieve consistent removal efficiencies.

To optimize and enhance the removal efficiencies of nitrogen, phosphorus, and sulfur in wastewater treatment, integrating additional processes alongside the membrane bioreactor (MBR) can significantly improve performance. Adding advanced oxidation processes (AOPs) like ozonation or UV/H_2_O_2_ after the MBR can effectively break down refractory organic compounds and reduce sulfur compounds [44]. Another effective method is ion exchange, which targets nitrogen compounds like ammonium and phosphorus by using ion exchange resins. This process is highly efficient in capturing these nutrients, thereby enhancing the overall nutrient removal efficiency and producing effluent that meets stringent discharge standards [45]. Additionally, membrane filtration processes such as nanofiltration (NF) and reverse osmosis (RO) serve as tertiary polishing steps. These technologies are adept at removing fine particulates, dissolved salts, and organic compounds, producing high-quality effluent. RO, in particular, excels in removing dissolved nitrogen and phosphorus compounds, as well as sulfate ions, by forcing water through a semipermeable membrane, achieving the highest level of contaminant removal suitable for various reuse applications or safe discharge into sensitive environments [46]. These aspects can be considered as future research directions of this study.

### 3.3. Influence of Organic Loading on Membrane Fouling and Backwashing Efficiency

#### 3.3.1. Membrane Flux and Transmembrane Pressure (TMP)

In our investigation, we assessed membrane fouling by tracking the evolution of TMP and flux over time, adapted to our operational conditions. Within Phase 1, the anaerobic membrane experienced an organic loading rate of 0.58 ± 0.07 kg COD/m^3^/day, which corresponded to a TMP of 42 ± 4 kPa and a flux of 25 ± 4 LMH, reflecting initial adaptation to the wastewater constituents. As the system transitioned to Phase 2, with increased loading to 3.24 ± 1.11 kg COD/m^3^/day, there was a noticeable increase in TMP to 58 ± 3 kPa, and a slight decline in flux to 22 ± 3 LMH (Table 3). This trend indicates the onset of fouling processes, likely due to a heightened formation of a cake layer on the membrane surface, a phenomenon commonly observed in the absence of effective fouling mitigation strategies within those phases [47].

By Phase 4, despite the further increased organic loading rate of 8.11 ± 2.03 kg COD/m^3^/day, the TMP increment was marginal, reaching 73 ± 4 kPa, and the flux saw a modest reduction to 21 ± 4 LMH. This phase’s data suggest that the fouling layer’s growth could be reaching a plateau, potentially due to the limits of cake-layer compression or a balance between fouling and cleaning forces within the system [48]. In Phase 4, the anaerobic membrane sustained an organic loading of 7.39 ± 0.95 kg COD/m^3^/day, with the TMP marginally rising to 76 ± 5 kPa and flux stabilizing at 20 ± 3 LMH. This stabilization could be attributed to the efficiency of the in situ membrane cleaning protocols applied, such as periodic backwashing, which seems to control the fouling without leading to significant loss in permeability [49]. The oxic membrane, subjected to a lower organic loading rate throughout the phases (0.42 ± 0.11 to 4.32 ± 0.88 kg COD/m^3^/day), demonstrated a more subdued TMP increase from 24 ± 2 kPa to 30 ± 3 kPa, and the flux varied between 13 ± 2 and 9 ± 3 LMH. The relatively lower fouling rate in the oxic membrane phases suggests the efficacy of aerobic microbial activity in maintaining membrane performance, potentially by the air scouring available in the reactor and the metabolizing and reducing of the organics that contribute to fouling [50].

#### 3.3.2. SEM-EDS Analysis of the Fouling Composition and Efficacy of Membrane Cleaning Strategies

The SEM-EDS analysis presented a detailed characterization of the fouling on the membranes. Morphological examination showed a light-brown, moist, and creamy layer indicative of organic matter with embedded rod-shaped bacteria, suggesting biofouling and biofilm formation [51] (see Supplementary Materials). The corresponding EDS mapping is expected to display elevated levels of carbon and nitrogen, suggesting the presence of organic biomass, and possibly higher sulfur content, hinting at the activity of sulfate-reducing bacteria [52]. Conversely, the aerobic membrane SEM images suggest a less dense fouling with more varied structures, indicative of the oxidative breakdown of organic matter facilitated by the presence of oxygen. The EDS mapping for the aerobic membrane may show reduced levels of carbon and nitrogen, reflecting more effective degradation of organic compounds. The analysis illustrated a distinct fouling structure, with organic components primarily forming the outer layer, while inorganic compounds contributed to a complex matrix, integrating with the biological elements to form a cohesive fouling layer. The SEM-EDS analysis of cleaned states of fouled membranes provided detailed insights into the inorganic fouling characteristics and the efficacy of different cleaning strategies (Table 4). For the anaerobic membrane, carbon and nitrogen showed high weight percentages in the fouled state, at 42.9% and 41.0%, respectively. After cleaning with permeate, the percentage of these elements significantly decreased, indicating the removal of organic foulants. Notably, the application of citric acid resulted in the lowest carbon content (11.2%), suggesting an effective dissolution of organic compounds. Similarly, citric acid cleaning significantly reduced the nitrogen content to 80.1%, which may indicate the removal of nitrogenous compounds often associated with microbial activity and proteinaceous substances [53].

Phosphorus, sulfur, and calcium showed reductions during postcleaning, which aligns with previous studies indicating the formation of inorganic precipitates, such as struvite and vivianite, in membrane systems [54]. The organic pollutants have ionizable groups, such as carboxyl groups (COO−), which can precipitate the fouling layer’s Ca^2+^, Mg^2+^, and Fe^3+^. Cations like Ca^2+^ and Mg^2+^ have the ability to stick to organic foulants in the fouling layer, like the humic acid group functional (COO−). The adhesion force increased in this order: Ca^2+^ > Mg^2+^, with calcium showing the greatest ability to bind to the (COO−) group functional of foulants in the fouling layer [55].

The aerobic membrane’s analysis revealed a similar trend, with a postcleaning decrease in the presence of phosphorus, sulfur, and silicon, elements commonly associated with biological and colloidal fouling. The reduction in calcium content postcleaning with citric acid further confirms its efficacy, supported by the literature, which recognizes citric acid’s ability to disrupt scales containing calcium and other inorganic compounds. These findings suggest that NaOCl cleaning primarily targets organic fouling, while citric acid is more effective for inorganic fouling, a conclusion supported by other research findings [55,56].

#### 3.3.3. Three-Dimensional Fluorescence Excitation–Emission Matrices Analysis (3D-EEM)

**Figure 4:**
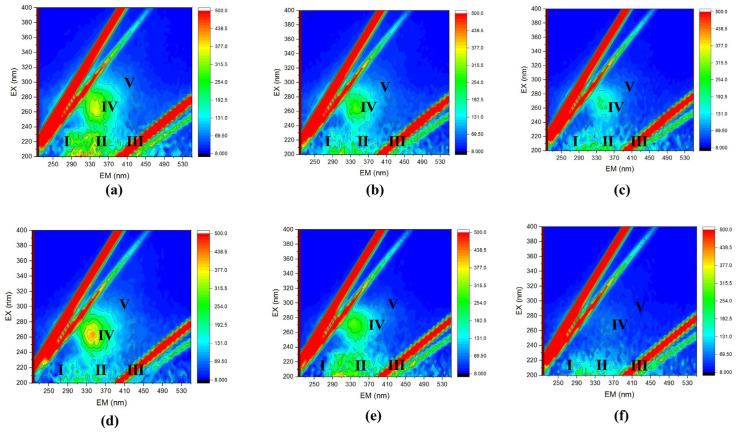
Fluorescence excitation and emission matrix of the membrane cleaning solutions: (**a**) permeate cleaning solution for An Mem; (**b**) NaOCl cleaning solution for An mem; (**c**) citric acid cleaning solution for An mem; (**d**) permeate cleaning solution for Ox Mem; (**e**) NaOCl cleaning solution for Ox mem; (**f**) citric acid cleaning solution for Ox mem. Region I: tyrosine-like proteins; Region II: tryptophan-like protein; Region III: fulvic acid-like (FA) substances; Region IV: soluble microbial by-product-like substances; Region V: humic acid-like (HA) substances.

In the evaluation of membrane-cleaning efficacy, 3D-EEM fluorescence spectroscopy revealed distinct patterns of organic and inorganic compound removal across the utilized cleaning solutions. For the anaerobic membrane, permeate cleaning exhibited the presence of tyrosine-like aromatics (Region I) and proteinlike substances (Region II), indicating a moderate removal efficiency and persistence of organic matter (Figure 4). Sodium hypochlorite (NaOCl) cleaning displayed a substantial reduction in these regions, underscoring its oxidative strength and effective biofouling mitigation. Citric acid cleaning emerged as the most effective, with a significant diminution in regions indicative of humic substances (Region V), suggesting its superiority in dislodging complex organic and inorganic fouling composites. The aerobic membrane cleaning analyses paralleled these findings; permeate cleaning achieved modest reductions in Regions II and IV, which denote proteinlike substances and microbial by-products, respectively. NaOCl treatment resulted in a notable decrement in humic acid-like substances (Region V), affirming its efficacy against entrenched organic foulants. However, citric acid cleaning outperformed other treatments, evidenced by the marked reduction across all regions, particularly in Region V, denoting a comprehensive cleaning action. The spectroscopy analysis thus delineates the specific efficacies of each cleaning agent, with citric acid demonstrating a superior capacity to purge both organic and inorganic substances from the membranes. NaOCl’s effectiveness was largely confined to the organic fraction, suggesting that a targeted or sequential cleaning strategy may enhance overall membrane restoration.

The FI of backwashing solutions on the anaerobic membrane also revealed the reduction of its value from 1.80, 1.67, and 1.20 with permeate cleaning, NaOCl cleaning, and citric acid cleaning solutions, respectively. At an FI value of 1.80, one would expect a substantial contribution of organic matter from microbial origin, which shows that these microbial material substances have been reduced in the membrane fouling layer with the cleaning steps. Also, at the aerobic membrane, FI values were observed at 1.45, 1.56, and 1.72 for permeate cleaning, NaOCl cleaning, and citric acid cleaning solutions, respectively. The permeate solution’s FI of 1.45 indicated effective removal of humic substances, characteristic of terrestrial organic matter. In contrast, the FI of 1.56 for NaOCl solution suggests a breakdown of these substances into simpler, microbial-like components. The citric acid solution’s FI of 1.72 points to a high presence of microbial organics, highlighting citric acid’s chelating efficiency in disrupting fouling complexes on the membrane [57].

### 3.4. Microbial Community Sucession

#### 3.4.1. Species Diversity and Community Structure

A richness and diversity analysis of the microbial community from anaerobic, anoxic, and oxic reactors provides a snapshot of microbial community diversity over time (Supplementary Materials). Diversity indices like ACE, Chao1, Shannon, and Simpson indicate species richness and evenness. For the anaerobic reactor, initial diversity is high, suggesting a rich and varied microbial community. Over time, species richness appears to stabilize, indicating that the community may have reached equilibrium within the reactor conditions. In the anoxic reactor, there is a dip in diversity on Day 62, which could indicate a disturbance or shift in the microbial community, but it recovers by Day 90, suggesting resilience or adaptation of the community to the reactor conditions. The oxic reactor shows high diversity, maintained over time, which is consistent with the need for a diverse range of functions and metabolic activities necessary for effective organic matter degradation in the presence of oxygen. High coverage values across all days and reactors imply that the majority of the microbial community is being captured in these samples, ensuring that the diversity indices are representative of the actual community structure [58].

The microbial community within the anaerobic reactor shows (Supplementary Materials) a high abundance of Thermoplasmatota (increasing from 5.15% to 11.24%) and *Firmicutes* (increasing from 16.61% to 24.88%), indicating robust anaerobic digestion and potential sulfate-reducing activities [59]. Proteobacteria, starting at 19.20% and spiking to 25.54%, point towards diverse metabolic functions, including sulfate reduction and denitrification. Desulfobacterota, ranging from 9.51% to 5.64%, are also key for sulfur cycling, which aligns with observed sulfammox and SDAD processes [60]. In the anoxic reactor, *Proteobacteria* dominate (33.95% by Day 220) along with Bacteroidota (18.04% by Day 220), suggesting ongoing denitrification processes [33]. The notable presence of Deinococcota, which significantly increases to 34.18%, could indicate resistance to oxidative stresses and a role in the transformation of organic compounds. The oxic reactor, dominated by *Proteobacteria* (63.37% by Day 220) and *Bacteroidota* (8.83% by Day 220), suggests active nitrification and organic matter degradation. The presence of *Deinococcota* and *Planctomycetota*, which fluctuate throughout the study period, may reflect the roles in nutrient removal through nitrification and other aerobic processes [33].

#### 3.4.2. Microbial–Physicochemical Interplay in An MBR-Anoxic/Oxic MBR System

**Figure 5:**
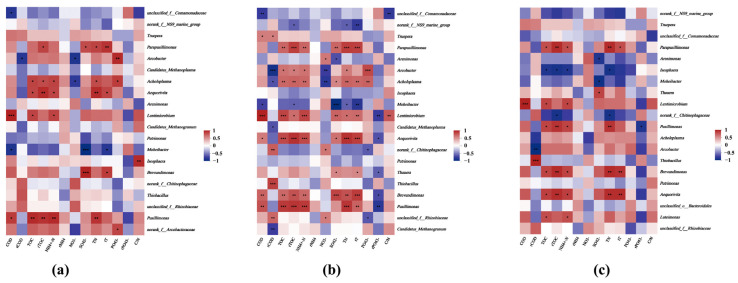
Spearman correlation heatmaps of (**a**) anaerobic; (**b**) anoxic; and (**c**) oxic reactors, corelating environmental factors and genus-level species (rCOD, rTOC, rNH4, rPO_4_^3−^, and C/N represent the removal efficiencies of COD, TOC, ammonia, total nitrogen, phosphate, and COD/TN ratio, respectively). The * denote statistical significance levels: * *p* < 0.05, ** *p* < 0.01, *** *p* < 0.001.

The Spearman correlation heatmap shows that genera such as *Candidatus Methanoplasma* and *Thiobacillus* have a strong positive correlation with sulfate removal efficiency, indicative of their roles in the sulfammox process and sulfur cycling in the anaerobic reactor. Furthermore, these genera also show correlations with the removal of COD and ammonia, reflecting their involvement in broader metabolic processes such as methane production and nitrogen cycling within the system (Figure 5). The observed correlation of *Thiobacillus* with sulfate indicates its potential role in the anoxic reactor’s sulfur transformation, likely through the SDAD process. Additionally, the positive correlations of genera like *Thauera* with nitrate and nitrite levels highlight their contribution to conventional denitrification. *Thiobacillus* in the oxic reactor, correlating positively with sulfate reduction, indicates its involvement in sulfur oxidation. This genus is key in the sulfur cycle, converting reduced sulfur compounds to sulfate. Additionally, genera like *Truepera* and *Parapusillimonas*, positively correlated with COD and TOC removal efficiencies, may contribute to the breakdown of complex organic compounds [61,62]. This multifaceted microbial interaction is crucial for the system’s efficacy in pollutant transformation and removal.

### 3.5. Improved Mechanism Analysis at the Gene Level

#### 3.5.1. Key Genes for Nitrogen Removal

**Figure 6:**
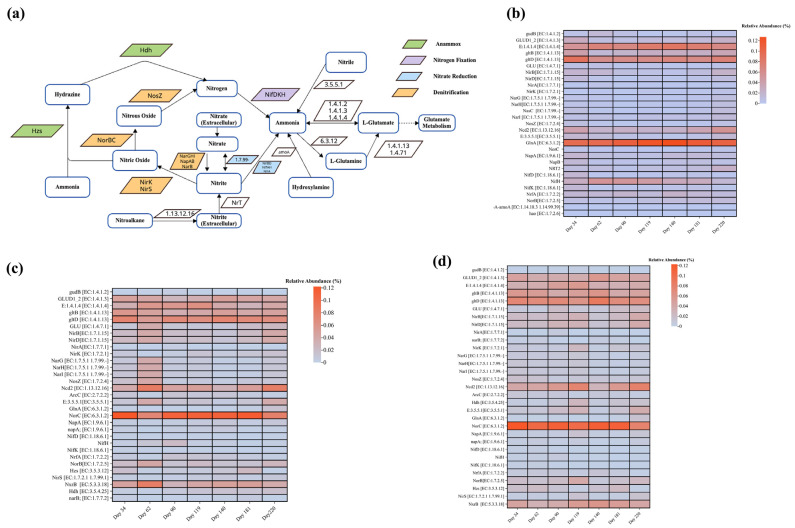
Enzyme and gene abundance of key metabolic pathways of reactors: (**a**) N cycle and relative abundance of enzymes involved in N cycle; (**b**) anaerobic reactor; (**c**) anoxic reactor; (**d**) oxic reactor.

The anaerobic reactor’s genetic profile, as showcased in Figure 6, reflects an evolving microbial environment adept at nitrogen compound transformation. Early stages hint at methanogenesis with genes like *NirK* gaining prominence, indicative of the burgeoning SDAD process. This is in line with the increased *NorB* activity, suggesting an active denitrification pathway. Crucially, the variable presence of the *pmoA-amoA* complex points to a responsive sulfammox process tailored to the reactor’s conditions. Nitrite reduction-related enzymes *NirB*, *NirD*, and *NirA* and nitrate-reducing enzymes *NarG*, *NarH*, *NasC*, and *NarI* exhibit varied abundances, indicating possible participation in conventional denitrification as well. These fluctuations underscore a microbial community dynamically attuned to the complex wastewater matrix, showcasing a preference for methanogenic and denitrification pathways over time, which is supported by the work of Huang et al. and Zhang et al. [18,63].

As the conditions within the anoxic reactor evolve, the enzyme profile undergoes a marked shift. *NirK* and *NorB* enzymes reflect a clear trend towards a more mature SDAD process, while the *pmoA-amoA* complex’s fluctuations suggest a fine-tuning of the sulfammox pathway. *NosZ*’s rising prominence by Phase 3 parallels a robust denitrification process, painting a picture of a microbial community that starts with a broad nitrogen removal strategy and gradually hones into SDAD and conventional denitrification mechanisms.

The oxic reactor’s heatmap data, illustrated in Figure 6, depict a microbial consortium increasingly adept at managing nitrogen compounds. The transition from the low enzyme prevalence of Phase 1 to the heightened activity in Phase 3 suggests a significant ramp-up in nitrification, underscored by the increased detection of *Hao* [62]. This phase-wise intensification suggests not just an acclimatization to aerobic conditions but an enhancement of nitrification capabilities over time.

#### 3.5.2. Key Genes for Sulfur Removal

**Figure 7:**
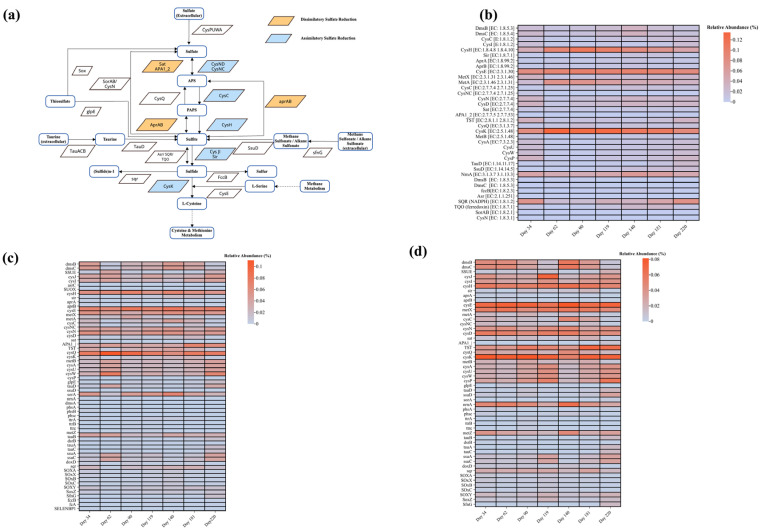
Enzyme and gene abundance of key metabolic pathways of reactors: (**a**) S cycle and relative abundance of enzymes involved in S cycle; (**b**) anaerobic reactor; (**c**) anoxic reactor; (**d**) oxic reactor.

The increasing abundance of the *CysH* enzyme in the anaerobic reactor in the early days mirrors the microbial community’s adeptness at sulfammox activities (Figure 7). It reveals an early-stage dominance in the microbial ability to initiate sulfur metabolism as a response to the introduction of sulfur-bearing wastewater. Sulfate adenylyltransferase (*Sat*) activities underscore the continual presence and likely dominance of sulfate-reducing bacteria, which form the cornerstone of the anaerobic sulfur cycle [63].

During the acclimation phase in the anoxic reactor, enzymes related to the sulfur cycle start from a baseline level, increasing over time as the microbial community adjusts to the lack of oxygen while exploiting sulfate and other sulfur compounds present in the wastewater. The enzymes involved in dissimilatory sulfate reduction, like *Sat* and *AprAB*, signal an active engagement in the sulfur cycle [64], highlighting a period of functional diversification and possibly signaling competitive or synergistic interactions between sulfur-oxidizing bacteria (SOB) and SRB.

The heatmap analysis for the oxic reactor outlines the evolution of the sulfur cycle within this oxygen-rich environment. The relatively higher abundance of enzymes like *SoxA*, *SoxB*, *SoxC*, *SoxX*, *SoxY*, and *SoxZ* across various time points signifies the occurrence of sulfur oxidation processes. These enzymes are part of the Sox enzyme system responsible for the oxidation of reduced sulfur compounds to sulfate, which aligns with the aerobic nature of the reactor. Notable is the presence of enzymes like *sqr* (sulfide:quinone oxidoreductase) and *SoeABC*, which are involved in the oxidation of sulfide to sulfate, suggesting that the sulfur cycle is active.

This systematic enzymatic cascade ensures that each stage of the reactor network contributes effectively to the holistic management of nitrogen and sulfur, leading to the efficient breakdown and removal of sulfur and nitrogen compounds from the wastewater.

## 4. Conclusions

This study conclusively demonstrated the high efficiency of the integrated AnMBR and A/O MBR system in the treatment of rubber industry wastewater. The integrated system demonstrates exceptional performance, with overall organic matter removal efficiency exceeding 99.4% with an organic input of 7.39 ± 0.95 (kg COD/m^3^·day) and significant reductions in sulfate, ammonia nitrogen, and total phosphorus. The biological system eliminates 60–80% of TN and NH_3_-N overall. On average, this approach eliminates TP by 70.8%. Despite an average whole biological system removal of 50.6%, the anaerobic reactor eliminated 44.9% of the raw WW sulfur.

Anaerobic membrane fouling was characterized by the accumulation of organic matter and biofilm development, whereas aerobic membrane fouling exhibited decreased carbon and nitrogen levels, indicating more varied structures due to differential microbial activity and oxidation processes. The use of citric acid was particularly notable, showing great promise for effective fouling control within the system. The microbial community within the AnMBR-A/O MBR system undergoes evolution, enabling effective simultaneous sulfate reduction and nitrogen removal. The anaerobic stage is marked by sulfate-reducing bacteria like Desulfovibrio and *Thiobacillus*, which are essential for the initial reduction of sulfate, a critical step in effluent detoxification. As the wastewater flows into the anoxic reactor, denitrifying bacteria take precedence, facilitating the conversion of nitrate into nitrogen gas, thereby enhancing nitrogen removal. In the final aerobic stage, the system capitalizes on nitrifying and sulfur-oxidizing bacteria, which efficiently convert ammonia into nitrate and manage sulfide levels. Throughout these stages, enzymatic activities such as those from hydrazine synthase and nitrate reductase are pivotal in catalyzing the transformations of nitrogen and sulfur compounds, showcasing the system’s versatile and adaptive approach to the treatment of rubber industrial wastewater. This innovative approach to wastewater treatment, combining both biological processes and membrane technology, could set a precedent for future sustainable water management solutions in rubber industry wastewater treatment.

## Figures and Tables

**Table 1:** Characteristics of pretreated natural rubber wastewater from centrifuged latex factory (*n* = 20) (average ± S.D.) [26].

Parameter	Influent to the Biological System (Pretreated Wastewater)
COD, mg/L	23,647 ± 3826
TOC, mg/L	7438 ± 1068
pH	6.75 ± 0.25
EC, mS/cm	24.55 ± 8.67
TSS, mg/L	1275 ± 45
NO_3_^−^-N, mg/L	113 ± 37
NO_2_^−^-N, mg/L	35.55 ± 20.18
NH_3_-N, mg/L	1545 ± 97
TN, mg/L	3529 ± 312
SO_4_^2−^-S, mg/L	425 ± 180.5
S^2−^, mg/L	8.32 ± 3.72
PO_4_^3−^-P, mg/L	156 ± 35
NH_3_^−^-N/SO_4_^2−^-S	3.635 ±1.561
COD/TN	6.701 ±1.235
TN/PO_4_^3−^-P	22.621 ±5.456
TN/SO_4_^2−^-S	8.304 ±3.602

**Table 2:** Detailed operational characteristics for the reactor system over 225 days (average ± S.D.).

Phase		1	2	3	4
Days		0–40	41–70	71–130	131–225
Pretreated influent (mg/L)	COD	1159 ± 136	6486 ± 2219	24,319 ± 6095	22,158 ± 2859
	NH_3_	720.7 ± 249.3	1511.6 ± 552.9	1559.9 ± 141.5	1537.3 ± 61.2
	SO_4_^2−^-S	258.0 ±2.8	260.0 ± 22.2	221.9 ±179.1	477.1 ± 169.2
	TN	1890.0 ± 28.3	1987.9 ± 389.9	3592.9 ± 443.5	3487.6 ± 195.7
	TP	23.6 ± 76.5	76.5 ± 38.3	156.1 ± 34.8	145.1 ± 22.7
An MBR effluent (mg/L)	COD	839 ± 224	2870 ± 724	10,284 ±4374	12,951 ±2633
	NH_3_	537.3 ±194.6	1324 ± 466.5	1362.7 ±195.2	1442.2 ± 48.7
	SO_4_^2−^-S	125 ± 7.1	163.5 ±41.1	119.3 ± 74.7	259.3 ± 97.5
	TN	1375.0 ± 77.8	1223.2 ± 215.7	2507.0 ± 774.1	3186.2 ± 164.2
	TP	20.9 ± 5.6	26.9 ± 9.8	44.4 ± 12.1	40.7 ± 12.7
Final effluent (mg/L)	COD	98.2 ± 26.9	253.6 ±113.8	655 ± 282	118 ± 74
	NH_4_^+^-N	210.6 ±125.6	244.3 ± 74.2	375.3 ±92.1	412.3 ± 92.4
	SO_4_^2−^-S	169.0 ± 28.3	202.8 ±15.2	181.6 ± 83.3	238.6 ± 118.6
	TN	1212.0 ± 31.1	1067.5 ± 261.4	826.2 ± 107.9	947.3 ± 227.6
	TP^−^	5.8 ± 3.3	6.2 ± 2.0	4.7 ± 4.8	2.2 ± 1.7
Avg. COD rem. eff. of the total system	(%)	91.5 ± 2.5	95.5 ± 1.1	97.2 ± 1.4	99.4 ± 0.4
Avg. NH_4_^+^-N rem. eff. of the total system	(%)	70.8 ± 7.6	83.1 ± 3.1	75.5 ± 6.1	72.9 ± 5.7
Avg. SO_4_^2−^-S rem. eff. of the total system	(%)	34.6 ± 10.2	21.8 ± 4.9	48.3 ± 10.8	49.6 ± 18.6
Avg. TN rem. eff. of the total system	(%)	35.9 ± 0.7	45.3 ± 14.3	76.4 ± 6.9	72.8 ± 6.6
Avg. TP rem. eff. of the total system	(%)	11.9 ± 8.8	61.3 ± 16.6	70.8 ± 8.8	71.3 ± 9.9

**Table 3:** Membrane filtration performance during operational stages.

Membrane	Parameter	Phase 1	Phase 2	Phase 3	Phase 4
Anaerobic membrane	Organic loading rate (kg COD/m^3^ d^−1^)	0.58 ± 0.07	3.24 ± 1.11	8.11 ± 2.03	7.39 ± 0.95
	TMP (kPa)	42 ± 4	58 ± 3	73 ± 4	76 ± 5
	Flux (LMH)	25 ± 4	22 ± 3	21 ± 4	20 ± 3
	Avg. permeability (L/m^2^·h·bar)	59.5	37.9	28.8	26.3
	Operating temperature	28.08 ± 0.80			
Oxic membrane	Organic loading rate (kg COD/m^3^ d^−1^)	0.42 ± 0.11	1.44 ± 0.36	3.43 ± 1.46	4.32 ± 0.88
	TMP (kPa)	24 ± 2	28 ± 6	31 ± 2	30 ± 3
	Flux (LMH)	13 ± 2	12 ± 2	10 ± 2	9 ± 3
	Avg. permeability (L/m^2^·h·bar)	54.2	42.9	32.3	30.0
	Operating temperature	27.74 ± 0.95			

**Table 4:** SEM-EDS composition of permeate-cleaned membrane, NaOCl-cleaned membrane, citric acid-cleaned membrane.

Element	Fouled Membrane Weight Percent of the Element (%)	Permeate-Cleaned Membrane Weight Percent of the Element (%)	NaOCl-Cleaned Membrane Weight Percent of the Element (%)	Citric Acid Cleaned-Membrane Weight Percent of the Element (%)	Minimum Detection Limit	Error (%)
Anaerobic Membrane						
C	42.9	16.9	24.3	11.2	1.7–4.9	12.8–25.1
N	41.0	74.1	64.3	80.1	1.4–1.5	12.2–14.3
P	4.5	1.4	2.4	1.3	0.2–0.5	7.1–25.2
S	0.6	0.1	0.1	0.2	0.2–0.5	19.2–68.9
Mg	0.1	2.3	1.0	3.0	0.2–0.5	8.8–36.1
Si	0.1	1.4	1.2	1.8	0.1–0.4	11.7–68.3
Ca	4.5	1.4	2.7	0.6	0.2–0.9	7.1–63.8
Fe	0.6	0.1	0.1	1.9	0.2–1.6	19.2–67.1
Aerobic Membrane						
C	43.1	45.8	11.4	8.0	0.4–2.8	11.4–28.2
N	51.8	49.3	80.8	83.0	0.8–1.1	11.2–13.2
P	0.8	1.0	0.9	0.9	0.2–0.4	11.8–32.5
S	0.4	0.4	0.1	0.1	0.2–0.4	24.8–70.8
Mg	0.3	0.4	2.9	3.4	0.1–0.4	8.1–49.7
Si	1.2	0.7	2.2	2.9	0.1–0.3	10.1–12.5
Ca	0.8	1.5	0.3	0.4	0.2–0.7	13.0–62.9
Fe	0.4	1.0	1.4	1.3	0.2–1.1	27.9–51.1

## Data Availability

Data are contained within the article or Supplementary Materials.

## Supplementary Materials

The following supporting information can be downloaded at: https://www.mdpi.com/article/10.3390/membranes14060130/s1, Table S1: Discharge standards for the centralized WWTP or for public sewers in China and Sri Lanka, Table S2. Cost of the conventional activated sludge, anaerobic MBR, and aerobic MBR systems, Figure S1. SEM images of the foulants of (a) anaerobic membrane, (b) aerobic membrane, Figure S2. EDS mapping of elements on the membrane’s outer surface (a) anaerobic membrane, (b) aerobic membrane, Table S3. Richness and diversity analysis of microbial community in the reactors (anaerobic, anoxic, oxic reactors), Table S4. Relative abundance percentage at phylum level in each reactor.
