# Supplementary material for: Enhancing Rubber Industry Wastewater Treatment through an Integrated AnMBR and A/O MBR System: Performance, Membrane Fouling Analysis, and Microbial Community Evolution
Source: Membranes (Basel). 2024 Jun 5;14(6):130. doi: 10.3390/membranes14060130 (PMC11205297; doi:10.3390/membranes14060130)
Supplement: Supplementary file 1 [file membranes-14-00130-s001.zip › membranes-3005556-supplementary.pdf]

Enhancing Rubber Industry Wastewater Treatment through Integrated AnMBR and A/O MBR system: Performance, Membrane Fouling Analysis, and Microbial Community Evolution

Table S1:Discharge standards for the centralized WWTP or for public sewers at China and Sri Lanka

Parameter	Sri Lanka [1]	China[2]
	Centralized treatment plant	Discharged to Public Sewers when secondary treatment is available
pH	5.5 – 9.0	6.5 – 9.5
Total Suspended Solids	500	400
Total Dissolved Solids (TDS)	3000	2000
Biochemical Oxygen Demand (BOD_5)	400	350
Chemical Oxygen Demand (COD)	800	500
Total Nitrogen (TN)	-	70
Ammoniacal Nitrogen (as N)	50	45
Sulfides (as S^{2-})	2.0	1.0
Sulfate (SO_4^{2-}-S)	350	600
Total Phosphate (as P)	3.5	8
Zn	5.0	5.0
Total Fe	-	10

(All parameters are in mg/L except for pH)

Table S2 : Cost of the Conventional Activated Sludge, Anaerobic MBR and Aerobic MBR systems [3,4]

System	Capital Cost (€/m^3)	O&M Cost (€/m^3)	Total Cost (€/m^3)	Total Cost (100% Farmland) (€ per m^3)	Total Cost (100% Landfilling) (€ per m^3)	Total Cost (100% Incineration) (€ per m^3)
CAS	0.59	0.66	1.25	1.25	1.32	1.40
ArMBR	0.62	0.70	1.32	1.29	1.36	1.40
AnMBR +ArMBR	0.66	0.74	1.40	1.36	1.43	1.47
AnMBR +ArMBRMethDN	0.70	0.77	1.47	1.43	1.51	1.54
AnMBR + CAS	0.74	0.81	1.54	1.51	1.58	1.62
AnMBR + CASMethDN	0.77	0.85	1.62	1.58	1.65	1.69

AnMBR – Anaerobic Membrane Bioreactor

ArMBR – Aerobic Membrane Bioreactor

CAS – Conventional Activated Sludge System

MethDN - Dissolved methane was used for energy production and denitrification.

(a)

(b)

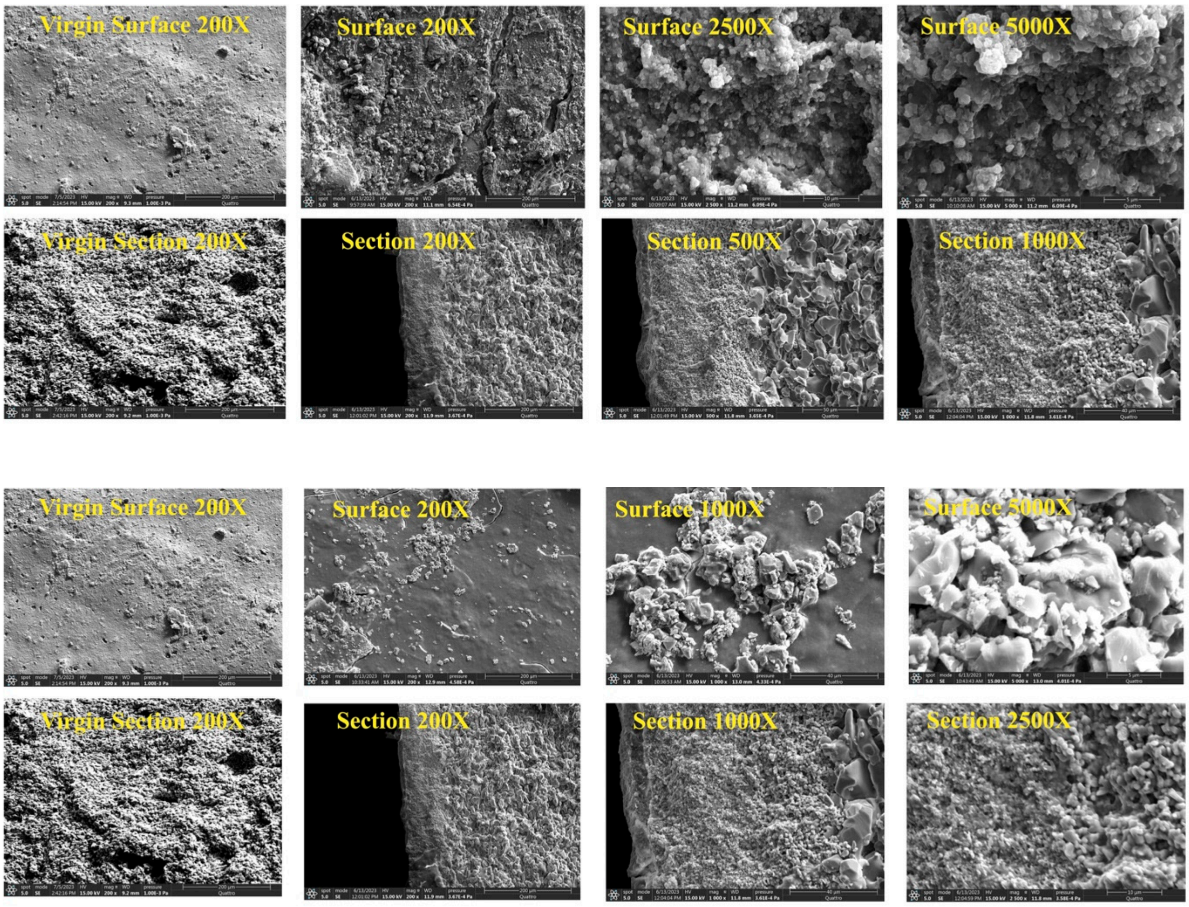

Figure S1. SEM images of the foulants of (a) anaerobic membrane, (b) aerobic membrane (Note: The inserted number is the magnification factor and both surface and section images are shown)

(a)

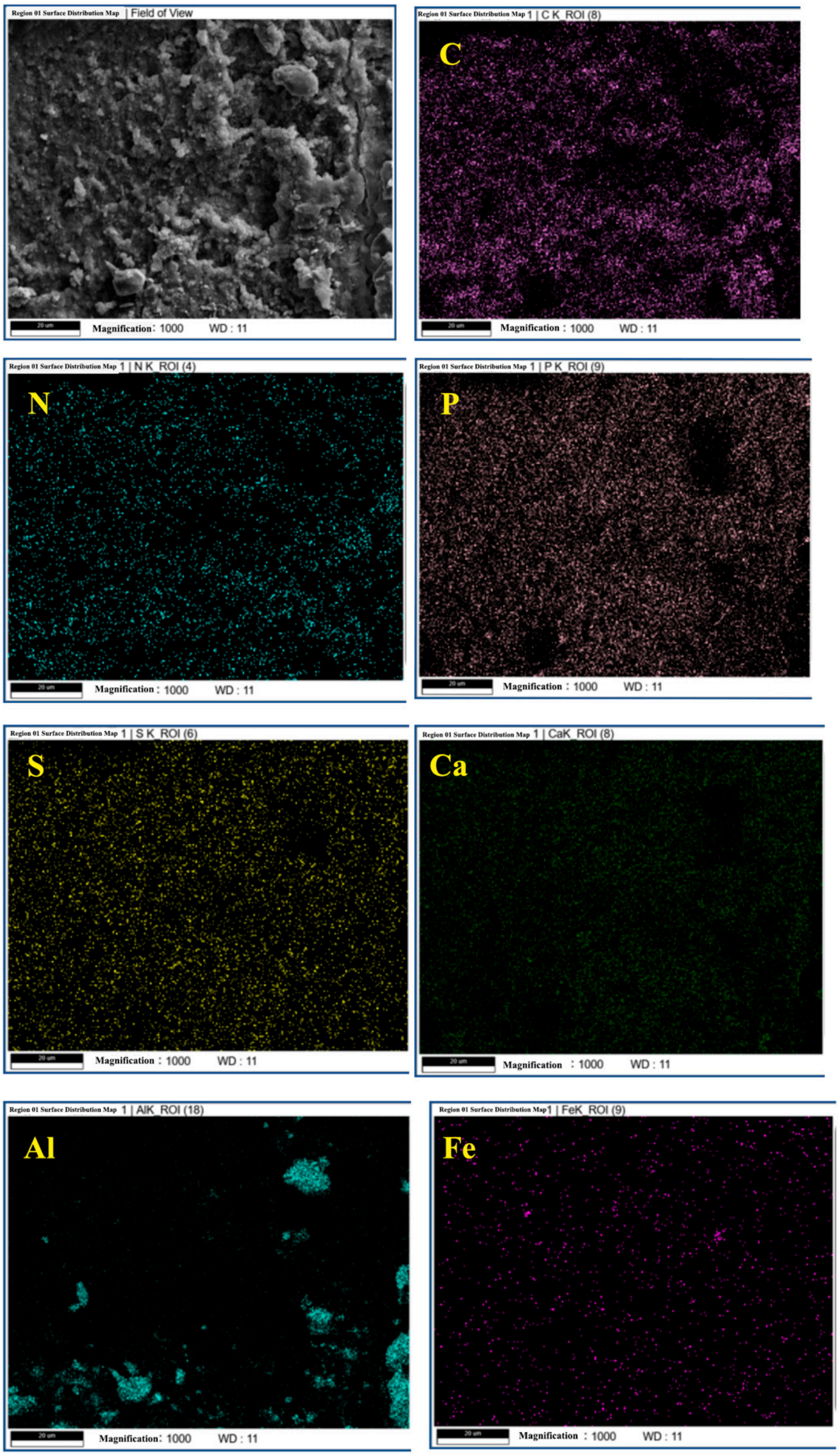
Region 01 Surface Distribution Map | Field of View
Magnification: 1000 WD : 11
Region 01 Surface Distribution Map 1 | C K_ROI (8)
C
Magnification : 1000 WD : 11
Region 01 Surface Distribution Map 1 | N K_ROI (4)
N
Magnification : 1000 WD : 11
Region 01 Surface Distribution Map 1 | P K_ROI (9)
P
Magnification : 1000 WD : 11
Region 01 Surface Distribution Map 1 | S K_ROI (6)
S
Magnification : 1000 WD : 11
Region 01 Surface Distribution Map 1 | CaK_ROI (8)
Ca
Magnification : 1000 WD : 11
Region 01 Surface Distribution Map 1 | AlK_ROI (18)
Al
Magnification: 1000 WD : 11
Region 01 Surface Distribution Map 1 | FeK_ROI (9)
Fe
Magnification : 1000 WD : 11

(b)

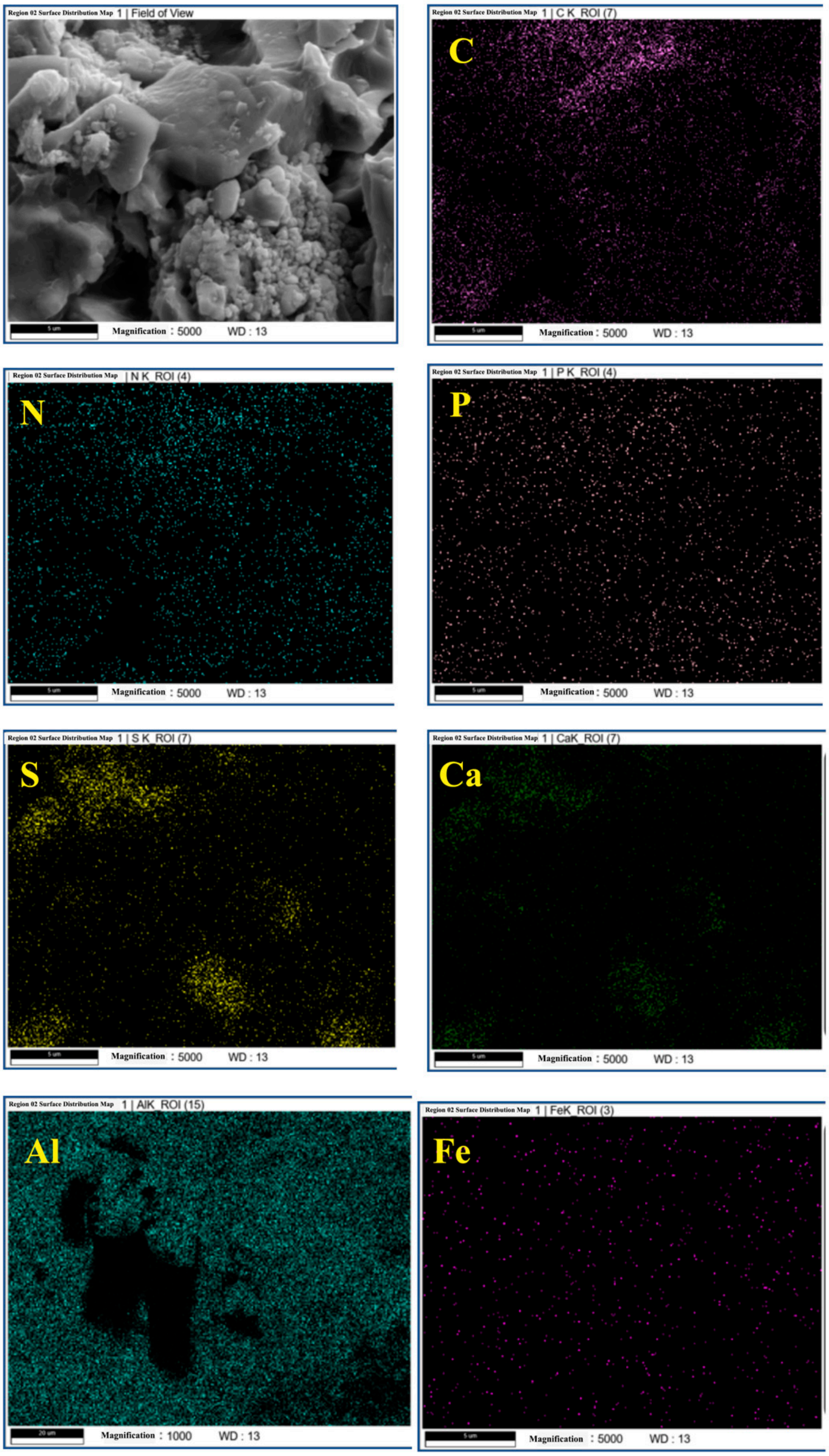

Figure S2. EDS-mapping of elements on the membrane's outer surface (a) anaerobic membrane, (b) aerobic membrane.

Table S3 Richness and diversity analysis of microbial community at the Reactor (Anaerobic, Anoxic, Oxic reactor)

Day	ACE	Chao1	shannon	simpson	coverage	sobs
			Anaerobic Reactor			
34	710.968947	699.594059	4.183512	0.039003	0.998722	679
62	525.09419	521.267857	3.399347	0.135513	0.999469	515
90	610.347411	601.5	3.557581	0.091867	0.999095	590
119	844.459657	843.235955	4.0943	0.086998	0.998368	805
140	873.756188	854.419847	3.927422	0.072107	0.99762	799
181	796.388311	787.179245	4.207034	0.054106	0.998525	761
220	808.038946	798.428571	4.827935	0.020086	0.998702	778
			Anoxic Reactor			
34	652.981294	646.881579	4.072234	0.055906	0.998781	622
62	393.707678	388.28	2.473228	0.298607	0.999626	386
90	840.956289	838.318182	4.864414	0.024101	0.999646	836
140	540.136663	537.101695	3.765696	0.085812	0.998899	511
119	644.85741	636.038462	4.536454	0.022584	0.998918	617
181	459.813919	456.542373	3.844413	0.064084	0.999725	455
220	472.565511	467.637931	3.189643	0.099072	0.999076	449
			Oxic Reactor			
34	623.609207	618.323944	4.142061	0.04895	0.999135	622
62	545.525366	542.052632	4.196707	0.042527	0.999567	386
90	810.131914	807.75	4.953558	0.02194	0.999705	836
119	501.782039	496.924242	3.965633	0.044993	0.999489	511
140	466.647174	459.84375	3.535252	0.115233	0.999292	617
181	397	397	3.769989	0.06051	1	455
220	519.437779	514.681818	3.377486	0.085982	0.999095	449

Table S4. Relative abundance percentage at Phylum level at each reactor

Type	Day 34	Day 62	Day 90	Day 119	Day 140	Day 180	Day 220
			Anaerobic Reactor				
Thermoplasmatota	5.1465	40.1868	43.4159	33.0541	19.8505	18.5270	11.2350
Firmicutes	16.6136	29.6244	15.5516	20.0826	21.1740	26.7611	24.8830
Bacteroidota	14.3854	9.1150	16.7807	16.1121	20.6254	13.7561	16.6234
Proteobacteria	19.2035	5.7129	7.0521	7.1839	4.3382	9.8073	25.5398
Campilobacterota	15.6578	3.6618	4.5093	8.3717	19.2881	20.3324	7.6480
Desulfobacterota	9.5143	4.8614	3.8820	5.8840	7.8682	5.8938	5.6382
Spirochaetota	3.0088	3.0147	5.5890	6.0806	2.8358	1.9784	3.2665
Thermotogota	9.7463	0.7788	0.2104	0.0846	0.0275	0.0079	0.0138
Synergistota	0.9361	1.0049	0.6608	0.7709	1.8014	0.2242	0.6372

Actinobacteriota	0.5192	1.3491	0.8437	0.7099	0.4189	0.1278	0.8161
others	5.2684	0.6903	1.5044	1.6657	1.7719	2.5841	3.6991
				Anoxic Reactor			
Proteobacteria	31.829	60.269	18.488	29.497	13.805	13.805	33.945
Bacteroidota	42.802	12.303	22.704	32.710	38.800	38.800	18.039
Firmicutes	4.450	7.762	13.054	15.538	8.818	8.818	3.158
Deinococcota	3.290	0.008	1.554	0.861	12.757	12.757	34.181
Thermoplasmatota	0.116	14.051	18.020	1.892	2.252	2.252	0.368
Campilobacterota	0.024	0.875	2.678	9.204	11.217	11.217	0.301
Planctomycetota	4.680	0.348	9.980	0.543	0.940	0.940	0.924
Desulfobacterota	0.047	1.434	2.232	2.163	2.203	2.203	0.128
Spirochaetota	0.069	0.936	4.391	2.269	1.335	1.335	0.022
Chloroflexi	4.128	0.521	2.126	0.374	0.765	0.765	1.290
others	8.564	1.493	4.773	4.950	7.107	7.107	7.644
				Oxic Reactor			
Proteobacteria	33.384	27.809	24.419	56.161	15.459	34.071	63.373
Bacteroidota	40.726	43.009	20.146	16.431	52.606	18.059	8.834
Firmicutes	3.770	2.930	12.260	9.693	4.545	3.202	14.378
Deinococcota	3.652	0.208	3.266	1.182	14.616	34.087	1.158
Planctomycetota	4.183	3.259	20.635	0.185	0.989	0.531	0.380
Campilobacterota	0.006	5.036	3.019	9.257	0.692	0.118	1.980
Chloroflexi	4.873	4.930	4.474	0.132	1.280	1.036	0.419
Thermoplasmatota	0.035	0.057	4.531	1.748	0.262	0.028	1.078
Myxococcota	2.834	1.613	0.643	0.159	5.182	1.272	0.126
Actinobacteriota	0.840	2.977	1.355	0.899	1.554	2.423	0.722
others	5.697	8.171	5.253	4.153	2.816	5.174	7.554

Reference:

1. Board of Investment of Sri Lanka *Environmental Norms*; 2011;
2. Water Quality Standards for Sewage Discharged into Urban Sewers *(GB/T 31962-2015)*; 2015;
3. Pretel, R.; Robles, A.; Ruano, M. V.; Seco, A.; Ferrer, J. Economic and Environmental Sustainability of Submerged Anaerobic MBR-Based (AnMBR-Based) Technology as Compared to Aerobic-Based Technologies for Moderate-/High-Loaded Urban Wastewater Treatment. *J. Environ. Manage.* **2016**, *166*, 45–54, doi:10.1016/j.jenvman.2015.10.004.
4. Gao, T.; Xiao, K.; Zhang, J.; Zhang, X.; Wang, X.; Liang, S.; Sun, J.; Meng, F.; Huang, X. Cost-Benefit Analysis and Technical Efficiency Evaluation of Full-Scale Membrane Bioreactors for Wastewater Treatment Using Economic Approaches. *J. Clean. Prod.* **2021**, *301*, 126984, doi:10.1016/j.jclepro.2021.126984.
